# Factors Associated with Suboptimal Control of Asthma among Adult Asthma Patients: A Cross-sectional Study

**DOI:** 10.2174/1874306402115010035

**Published:** 2021-07-07

**Authors:** Kuol Peter Lual, Mengist Awoke Yizengaw

**Affiliations:** 1 Mega Pharmaceutical Company Limited, Juba, South Sudan; 2 School of Pharmacy, Institute of Health Science, Jimma University, Jimma, Ethiopia

**Keywords:** Asthma, Suboptimal asthma control, Associated factors, Adults, JMC, Ethiopia

## Abstract

**Introduction:**

Asthma is a major public health problem that negatively impacts patients, families, and the community. Identifying risk factors for poor asthma control may greatly enhance the establishment of more effective treatment of asthma. The level of asthma control and risk factors for poor asthma control is relatively unknown in Ethiopia.

**Methods:**

A cross-sectional study was conducted on 150 adult asthma patients at the Outpatient Department (OPD) chest clinic of Jimma Medical Center (JMC), from February 15 –March 20, 2019. The Statistical Package for Social Science (SPSS) 21.0 was used for data analysis. Multivariate logistic regression was conducted to analyze the potential associated factors of suboptimal control of asthma.

**Results and Discussion:**

Of 150 adults diagnosed with asthma recruited in this study, 81 [54.0%] of them were females, and the mean age of the patients was 41.1 ± 12.4 years. Inhaled corticosteroid (ICS) plus short-acting beta-agonist (SABA) (64, 42.7%) was the most frequently used anti-asthmatic medication. Over one-fourth (26.0%) (95% CI, 19.2-33.8) of study participants had suboptimal asthma control. On multivariate logistic regression, being an urban dweller (AOR=3.70, p=0.025) and not applying proper inhalation technique (AOR=16.23, p=0.022) were increased the risk of suboptimal asthma control, while non-prescription anti-asthmatic drugs taking habit (AOR=0.25, p=0.010) reduces the odds of having suboptimal asthma control.

**Conclusion:**

Suboptimal asthma control is high among adult asthma patients. Being an urban dweller and not applying proper inhalation techniques were increased the likelihood of suboptimal asthma control, while non-prescription anti-asthmatic drugs taking habits had lower odds of suboptimal asthma control. The authors recommend large sample size studies on the comparative status of asthma control using prescription *versus* non-prescription anti-asthmatic medication.

## 1 INTRODUCTION

World Health Organization (WHO) defines asthma as one of the major non-communicable diseases. It is a chronic disease of the air passages of the lungs, which inflames and narrows them [1]. It is characterized by a history of respiratory symptoms, such as wheeze, shortness of breath, chest tightness, and cough that vary over time and intensity, together with variable expiratory airflow limitation [2]. The strongest risk factors for developing asthma are a combination of genetic predisposition with environmental exposure to inhaled substances and particles that may provoke allergic reactions or irritate the airways [1]. Global Initiative for Asthma (GINA) guidelines classified asthma into four categories based on disease severity: mild intermittent, mild persistent, moderate persistent, and severe persistent [2].

The Global Burden of Disease collaboration estimated that 420,000 people in the world died from asthma each year, *i.e*., more than 1000 per day [3]. Of this, over 80.0% of asthma-related deaths, as well as severe cases, occur in low and middle-income countries [1, 3]. Globally, asthma is ranked 16th among the leading causes of years lived with disability and 28^th^ among the leading causes of the burden of disease, as measured by disability-adjusted life years (DALYs). It accounts for the loss of over 15 million DALYs annually [4].

In Africa, the prevalence of asthma increased across all age groups; it accounted for 49.7 million (13.9%; 95% CI 9.6-18.3) among children <15 years, 102.9 million (13.8%; 95% CI 6.2-21.4) among people aged <45 years, and 119.3 million (12.8%; 95% CI 8.2-17.1) in the total population in 2010 [5]. In Ethiopia, the overall prevalence rate of asthma was 9.1% [6], and it is the 10^th^ Environmental burden by disease category [DALYs/1000 capita] per year with a rate of 1.2 [7].

Overall, asthma is a major public health problem that negatively impacts patients, their families, and the community by inducing work and school loss, poor quality of life, frequent emergency visits, hospitalizations, death [8] as well as increased health care expenditures that can include costs of office visits, hospitalizations, emergency room visits, tests, and medications [4].

Asthma is considered under good control when patients can avoid troublesome symptoms during the day and night, need little or no reliever medication, have a productive and physically active life, have normal or near-normal lung function, avoid serious asthma flare-ups (exacerbations or attacks) [2]. The control of asthma symptoms is a realistic goal, and studies have shown that achieving this in most asthma patients leads to a higher quality of life. Despite this, the control of asthma is generally poor [9]. European-wide, population-based surveys reported prevalence rates of unsatisfactory asthma control levels ranging from 56.6% [10] to 80.0% [11, 12].

**Fig. (1):**
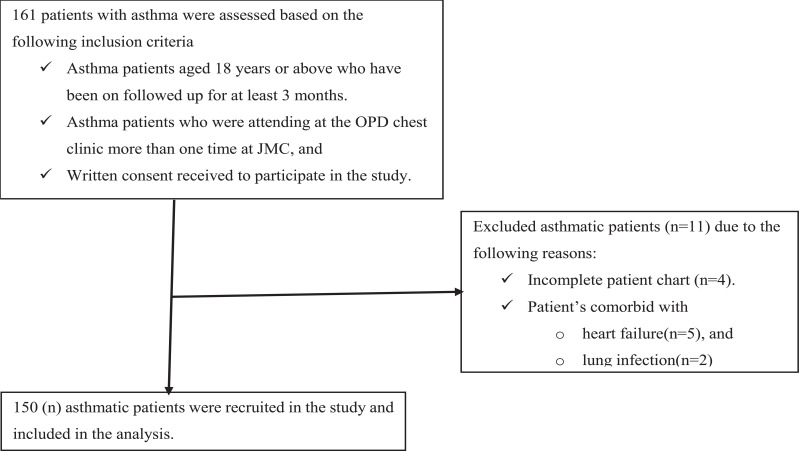
Patient flow chart.

Despite WHO's putting an objective on surveillance to map the magnitude of asthma and analyze its determinants of outcome [1], studies are still not sufficient, particularly in low-income countries [13]. Identifying risk factors for poor asthma control may greatly modify adverse effects and potentially lessen the financial burden of the disease. The level of asthma control and risk factors for poor asthma control is relatively unknown in Ethiopia [14]. Therefore, this study aimed to assess factors associated with suboptimal control of asthma among adult asthma patients at the chest clinic of the JMC (Fig. **1**).

## 2 MATERIALS AND METHODS

### 2.1 Study Design and Setting

A cross-sectional study was conducted at the OPD chest clinic of JMC, a tertiary teaching hospital in Jimma town, Jimma zone, Oromia, South-West Ethiopia. It is located 352 Km from Addis Ababa, the capital. The study was conducted from February 15 –March 20, 2019.

### 2.2 Sample Size Determination

The required sample size for the study was calculated by using the single population proportion formula by considering 76.1% prevalence of suboptimal asthma control in Ethiopia [14] and considering a 95% confidence level (α= 5%) with ±5% precision (d) and taking the estimated source population of 325 on follow up. The final sample size was 150 adult asthma patients on follow-up at the chest clinic of the JMC.

### 2.3 Data Collection Procedure and Quality Control

The data abstraction tool was developed to extract important variables by reviewing the patient charts and different kinds of literature that were used to assess the status of asthma control and its associated factors. Data were gathered by a data collector over 34 days (from February 15 –March 20, 2019), from the OPD chest clinic of JMC, from the patients' medical charts, and by interviewing patients for the respective information following a pre-test. The following data were retrieved from the patient medical chart- sex, anti-asthmatic medication, concurrent medication use, and comorbidities. Data extracted from interviewing the patient includes age, residency, educational status, marital status, job-status/category, OPD follow up status, without prescription medication-taking habit, smoking status, living status with a domestic animal, discontinuation of anti-asthmatic medication, the inhalation technique, and response towards assessing the status of asthma control on “daytime symptoms encountered more than twice/week,” “any night waking due to asthma,” “reliever needed more than twice/week,” and “any activity limitation due to asthma.”

### 2.4 Operational/Term Definitions

The suboptimal asthma control is considered [2] when in the past 4 weeks, has the patient had ≥1 of the following conditions occurred.

- Daytime symptoms more than twice/week.
- Waking up at due to asthma.
- Reliever needed more than twice/week.
- Any activity limitation due to asthma.

#### 2.4.1 Status of Smoking [15, 16]

- **Never:** An adult who has never smoked, or who has smoked less than 100 cigarettes in his or her lifetime, but who had quit smoking in the last 28 days.
- **Former (ex) smoker:** An adult who has smoked at least 100 cigarettes in his or her lifetime but who had quit smoking in the last 28 days.
- **Current smoker:** An adult who has smoked 100 cigarettes in his or her lifetime and has smoked in the last 28 days.

#### 2.4.2 Applying Proper Inhalation Technique

 Individuals are assessed on how they use the inhalation technique per the instructions of each device and considered proper if they follow all the procedures of inhalation.

#### 2.4.3 History of Discontinuing Medication

 History of the ceasing of anti-asthmatic medication by the patient without the clinician consults.

#### 2.4.4 Non-prescription Anti-asthmatic Medication Taking

 Patients taking the anti-asthmatic medication without a doctor's prescription [refilling anti-asthmatic medications without physician visit] [17].

List of anti-asthmatics medications used as an over – the – counter (OTC) in Ethiopia includes [18]:

(1) Ephedrine + Theophylline (in the form of Elixir (6mg + 30mg/5ml), Syrup (2.24% + 0.30%), and Tablet (11mg + 120mg),

(2) Salbutamol (Albuterol) (in the form of Oral Inhalation (aerosol)(0.1mg/dose), Syrup (2mg/5ml), and Tablet (2mg, 4mg, 4mg(s/r)), and

(3) Theophylline + Guaifenesin, 150mg + 90mg; 300mg +180mg (Capsule), 150mg + 90mg/15ml (Elixir), and 150mg + 90mg (Tablet).

#### 2.4.5 Residency [19, 20]

##### 2.4.5.1 Urban Dwellers

 People residing in areas, as localities with 2000 or more inhabitants that include all administrative capitals of regions, zones, and woreda’s, as well as localities with at least 1000 people who are primarily engaged in non-agricultural activities.

##### 2.4.5.2 Rural Dwellers:

 people residing in areas that are not classified as urban.

### 2.5 Data Analysis

The data were coded and entered into Epi data 4.2 then exported to the Statistical Package for Social Science (SPSS) 21.0 [21] for analysis. Descriptive analysis was conducted for baseline characteristics of categorical as well as continuous variables; frequency (%) and mean ± standard deviation (SD) were used to present the finding accordingly. Multivariate logistic regression analysis [reported with Adjusted Odd Ratios (AOR) with 95% Confidence Intervals (95% CI) was performed in a backward, step-wise manner, from an initial model which included all explanatory variables with a p-value of <0.25 on bivariate analysis, until a best-fit model was found, to identify factors independently associated with the suboptimal asthma control. *P*-value ≤0.05 on the final model was considered as a statistically significant associated factor for suboptimal asthma control.

## 3 RESULTS

### 3.1 Socio-demographic Characteristics of the Study Population

Of 150 adult asthma patients included in the study, 81 (54.0%) were females, and the mean (± SD) age of the patients was 41.13 ± 12.45 years. More than half of [52.0%] of the study population were rural dwellers (Table **1**).

### 3.2 Clinical Characteristics and Medications Used

Almost all patients (98.7%) had followed up at the OPD chest clinic for more than 1 year. More than one-third of the study participants were taking anti-asthmatic medications without any prescription, while 34.7% had a history of discontinuing the anti-asthmatic medication. One hundred thirty-six (90.7%) patients were reported accessing adequate anti-asthmatic drug information. Among the frequently used anti-asthmatic medications, ICS+SABA accounted highest (42.7%) followed by ICS+LABA (20.7%) and SABA+LAMA+ICS (14.7%). More than two-thirds (75.4%) of patients were taking a combination of two anti-asthmatic medications (Table **2**).

### 3.3 Status of Asthma Control and its Associated Factors

Of 150 adult asthma patients, thirty-nine (26.0%) (95% CI, 19.2%-33.8%) of them had suboptimal asthma control. On multivariate logistic regression, being an urban dweller (AOR=3.70, 95%CI, 1.18-11.59, P=0.025), and not applying proper inhalation technique (AOR=16.23, 95%CI, 1.48-177.76, P=0.022) were associated factors for suboptimal asthma control, while patients having a habit of taking non-prescription drugs (AOR=0.25, 95%CI, 0.08-0.71, P=0.010) had lower odds of suboptimal asthma control (Table **3**).

## 4 DISCUSSION

In the current study, 39 [26.0%] adult asthmatic patients had suboptimal asthma control. Being an urban dweller, non-prescription anti-asthmatic medication taking and not applying proper inhalation technique were associated factors of suboptimal asthma control.

Numerous studies across the world showed asthma control to be suboptimal. For instance, of asthma patients, in Europe 53.5% [22]-56.5% [23], United States of America 81.9% [24], Australia 45.7% [25], Thailand 46.5% [26], in Asia 65.3% [27], Europe-America 51.0% [28], Turkey 48.5% [29], and Tunisia 90.0% [30] had suboptimal asthma control. The suboptimal asthma control can be considered as the result of the interaction of the different variables, such as the role of guidelines diffusion and implementation, some disease-related factors (*i.e*., the presence of common comorbidities in asthma such as Eastroesophageal Reflux Disease (GERD), rhinitis, sleep disturbances and Obstructive Sleep Apnea (OSA) or patient-related factors (*i.e*., adherence to treatment and coping strategies) [13].

Despite, asthma control status in Africa has not been well studied, a recent study from Cameroun reported 42.0% of the study population had inadequately controlled asthma [31]. Other studies from Nigeria have reported 82.9% [32] inadequately controlled asthma. Democratic Republic of Congo (DRC) 56% [33], the North Africa countries 71.3% [34] and Ethiopia, Addis Ababa 53.3%-75.8% [14, 35] and Jimma 76.1% [36] have reported uncontrolled asthma. The results of the previous reports were higher than the current finding, which might be explained by the previous studies' findings that enrolled large sample size, studied over an extended duration along with different study settings. Therefore, all these factors might be considered favourable to increase the probability of detecting the actual status of asthma control. Another possible explanation might be that patients in the current study were recruited from a chest clinic located in a tertiary center (more pulmonologist avail); it has previously been demonstrated that asthma patients seen by specialists are more likely to be better managed than those followed by non-specialists [37, 38]. Furthermore, advancement in asthma treatment guidelines, as well as its diffusion and implementation over time may also contribute to improvements in asthma treatment outcomes [1, 39].

In the current study, all patients were taking more than one anti-asthmatic drug (combination therapy) and the most frequently used anti-asthmatic medications were ICS+SABA [42.7%] followed by ICS+LABA (20.7%) and SABA+LAMA+ICS (14.7%). However, they differed in comparison with other studies in India [40, 41]. Around 80-92% were taking multi-therapy (≥2drugs), and Methylxanthine (35.5-40%) was the most frequently prescribed drug among anti-asthmatics, followed by β2-agonists (23.9-27%), and corticosteroids (22.6-23%). This difference might be due to the current study conducted in the era of the updated GINA guideline 2019 avail, which recommends that all adults and adolescents with asthma should receive ICS-containing controller treatment to reduce their risk of serious exacerbations and to control symptoms [42]. Another possible reason might be due to differences in adherence to global asthma treatment guidelines across the study setup. On top of routine availability of short-acting bronchodilator anti-asthmatic medications (basically inhaled SABAs) in the current study environment, around one-third of the participants in the current study had the habit of taking non-prescription anti-asthmatic medication. These would justify the high proportion of taking ICS+SABA in this study. On the other hand, theophylline is not routinely recommended for the management of asthma in Ethiopia due to the safety profile [43].

We identified factors associated with suboptimal controlled asthma. Urban dweller asthmatic patients were more than threefold more likely to have suboptimal outcomes than rural dwellers (P=0.025). Exposures relating to environmental and lifestyle changes that originate from the urbanization process have been identified as risk factors for asthma as well as suboptimal control of it, including changes in diet, sedentarism, reductions in childhood infections, dust mites, high levels of vehicle, use of antibiotics, and environmental pollution [44].

Patients taking non-prescription drugs had 25% less likely to have suboptimal asthma control (P=0.010). Non-prescription anti-asthmatic drugs are not actively promoted. Despite this, nonprescription availability would primarily reduce economic barriers like transport cost, physician office visit cost, and better adherence to anti-asthmatic medication by increasing access to the medication [45, 46]. This will, in turn, reduce the probability of developing suboptimal asthma control (or enhance good asthma control).

In our study, asthma patients who were unable to apply the proper inhalation technique were more than sixteen-fold more likely to develop suboptimal outcomes (P=0.022). This might be explained by the proper inhalation technique that can increase medication efficacy, reducing both dose and side effects. On the other hand, deviation from the proper inhalation may result in a decrease in medication efficacy and increase side effects, affecting treatment outcome [47]. Despite the fruitful finding of our work, we acknowledge some limitations that are included in our study relying on physician-diagnosed asthma and the lack of incorporating spirometry-based assessment to assess the status of asthma control. The study also lacked a report on the severity classification of asthma and lacked data on specific non-prescription anti-asthmatic medications used. We were unable to construct a cause-effect relationship due to the cross-sectional study design. Furthermore, the study was single-center, which may limit the generalization of the results to the region and the country.

## CONCLUSION

In conclusion, suboptimal asthma control is high among adult asthma patients. Being an urban dweller and not applying proper inhalation techniques increased the likelihood of suboptimal asthma control, while patients having a habit of taking non-prescription drugs had lower odds of suboptimal asthma control. Clinicians/pharmacists have to pay attention to provide adequate anti-asthmatic drug information to their patients. We recommend large sample size studies on the comparative status of asthma control using prescription *versus* non-prescription anti-asthmatic medication.

## Figures and Tables

**Table 1:** Socio-demographic characteristics of the study population.

Variables		Frequency	Percentage
Sex	Male	69	46.0
	Female	81	54.0
Age	[Mean ± SD]	43.13 ± 12.45	years
Place of residency	Urban	72	48.0
	Rural	78	52.0
Marital status	Single	16	10.7
	Married	127	84.7
	Divorced	6	4.0
	Widowed	1	0.7
Educational status	Primary school	64	42.7
	Secondary school	8	5.3
	College and above	20	13.3
	Can’t write and read	58	38.7
Job-status	Unemployed	11	7.3
	Civil Servant	20	13.3
	Working at a non-governmental organization	7	4.7
	Merchant	14	9.3
	Farmer	78	52.0
	Labor worker	20	13.3

SD-Standard Deviation.

**Table 2:** Clinical-related factors among asthma patients.

Variables		Frequency	Percentage [%]
Comorbidity	Yes	3	2.0
	No	147	98.0
Follow up [>1 year]	Yes	148	98.7
	No	2	1.3
Non-prescription anti-asthmatic medication-taking habit	Yes	50	33.3
	No	100	66.0
Status of smoking	Never used	134	89.3
	Past smoker	12	8.0
	Current smoker	4	2.7
Live with a domestic animal	Yes	149	99.3
	No	1	0.7
History of discontinuing medication	Yes	52	34.7
	No	98	65.3
Adequate drug information	Yes	136	90.7
	No	14	9.3
Anti-asthmatic medications used	SABA+LABA	18	12.0
	ICS+SABA	64	42.7
	ICS+LABA	31	20.7
	SABA+LABA+ICS	15	10.0
	SABA+LAMA+ICS	22	14.6

SABA- Short-Acting Beta-Agonists, LABA-Long-Acting Beta-Agonists, ICS-Inhaled Corticosteroids, LAMA- Long-Acting Anti-Muscarinic Agent.

**Table 3:** Factors associated with the status of asthma control among adults with asthma.

Variables		Status of asthma control [N=150]		COR[95% CI]	p-value	AOR[95% CI]	p-value
		Good [n=111, 74.0%]	Suboptimal [n=39, 26.0%]				
Place of residency	Urban	50 [33.3]	22 [14.7]	1.57[0.75-3.29]	0.223	3.70[1.18-11.59]	0.025*
	Rural	61 [40.7]	17 [11.3]	1	-	1	-
Age in years [mean ± SD]	-	42.3±12.3	45.5±12.6	1.02[0.99-1.05]	0.175	1.035[.99-1.07]	0.054
Practicing non-prescription anti-asthmatic drugs taking	Yes	43 [28.9]	7 [4.7]	0.34[0.14-0.85]	0.021	0.25[0.08-0.71]	0.010*
	No	67 [45.0]	33 [21.5]	1	-	1	-
Smoking status	Past smoker	9 [6.0]	3 [2.0]	2.94[0.39-21.69]	0.290	-	-
	Current smoker	2 [1.3]	2 [1.3]	0.98[0.25-3.83]	0.977	-	-
	Never	100 [66.]	34 [22.7]	1	-	-	-
Discontinuation of anti-asthmatic medication	Yes	43 [28.7]	9 [6.0]	0.47[0.20-1.09]	0.081	0.37[0.14-0.98]	0.057
	No	68 [45.3]	30 [20.0]	1	-	1	-
Educational status	Primary school	42 [28.0]	22 [14.7]	4.71[1.00-22.19]	0.050	-	-
	Secondary school	6 [4.0]	2 [1.3]	3.00[0.34-26.19]	0.320	-	-
	Can’t write and read	45[30.0]	13 [8.7]	2.60[0.53-12.69]	0.238	-	-
	College and above	18[12.0]	2 [1.3]	1	-	-	-
Applying proper inhalation techniques	No	98 [65.3]	38 [25.3]	5.04[0.63-39.8]	0.125	16.23[1.48-177.76]	0.022*
	yes	13 [8.7]	1 [0.7]	1	-	1	-

AOR-Adjusted Odds Ratio, COR-Crude Odds Ratio, CI-Confidence Interval, SD-Standard Deviation *- statistically significant.

## LIST OF ABBREVIATIONS

COPD: = Chronic Obstructive Pulmonary Disease
DALYs: = Disability-Adjusted Life Years
DRC: = Democratic Republic of Congo
JMC: = Jimma Medical Center
GERD: = Gastroesophageal Reflux Disease
GINA: = Global Initiative for Asthma
ICS: = Inhaled Corticosteroids
LABA: = Long-Acting Beta-Agonists
LAMA: = Long-Acting anti-Muscarinic Agents
OPD: = Outpatient Department
OSA: = Obstructive Sleep Apnea
SABA: = Short-Acting Beta-Agonists
WHO: = World Health Organizations
